# Effect of soy on bone turn-over markers in men with type 2 diabetes and hypogonadism – a randomised controlled study

**DOI:** 10.1038/s41598-017-15402-9

**Published:** 2017-11-13

**Authors:** T. Sathyapalan, M. Aye, A. S. Rigby, W. D. Fraser, E. S. Kilpatrick, S. L. Atkin

**Affiliations:** 1 Academic Diabetes, Endocrinology and Metabolism, Hull York Medical School, University of Hull, Hull, UK; 2 Department of Academic Cardiology, University of Hull, Hull, UK; 3 Norwich Medical School, Faculty of Medicine and Health Sciences, University of East Anglia, Norwich, UK; 4 Department of Clinical Chemistry, Sidra Medical and Research Center, Doha, Qatar; 5 Weill Cornell Medical College Qatar, Education City, PO Box 24144 Doha, Qatar

## Abstract

Type 2 diabetes (T2DM) is associated with increased risk of fractures. Soy supplementation has been shown to have a beneficial effect on bone turnover markers (BTM) in postmenopausal women. However, the effect of soy supplementation on BTM in T2DM and particularly in men is unclear. We performed an analysis of a randomized double blind parallel study of 200 men with T2DM treated with soy, either with or without isoflavones. Outcome measures were type I collagen crosslinked beta C-telopeptide (βCTX), and type 1 procollagen-N-propeptide (P1NP). The men, with a total testosterone <12 nmol/L, were treated with 15 g soy protein containing 66 mg of isoflavones (SPI) or 15 g soy protein alone without isoflavones (SP) daily for three months. There was a 15% reduction in βCTX after three months of SPI compared to SP supplementation. There was no significant difference in P1NP with either SPI or SP supplementation. There was a significant linear correlation between the reduction in βCTX in the SPI group with the reduction in HbA1c (r^2^ = 0.42; p = 0.04) and HOMA-IR (r^2^ = 0.54; p = 0.02). Our study indicates that there was a significant reduction in bone resorption following 3 months of SPI supplementation that correlated with an improvement of glycemic control in men with T2DM.

## Introduction

There is substantial epidemiological evidence that people with T2DM has increased risk of fractures^1,2^. This increase in fracture risk occurs despite bone mineral density (BMD) being comparatively higher in those with T2DM than those without. In a meta-analysis, Vestergaard reported an increased Z-score of 0.41 at the spine and 0.27 at the hip associated with T2DM^2^. T2DM has been shown to be an independent risk factor for fractures, which is not attributable to increased body mass index (BMI) or other classical osteoporosis risk factors^3^.

Testosterone is anabolic for bone primarily through its aromatization to estrogen in adipose tissue^4^. Low testosterone in the setting of low or inappropriately normal luteinizing hormone and follicle-stimulating hormone is common in the diabetes population and may contribute to impaired bone strength in these men with T2DM^5^.

Production and consumption of soy foods within Western countries have increased dramatically with postulated health benefits including improvement in bone health, especially in women^6^. Habitual intake of soy isoflavones has also been associated with a reduced risk of T2DM and cardiovascular disease (CVD)^7^.

Bone turnover markers (BTMs) are biomarkers for fracture risk that have been used for the evaluation of effects of osteoporosis treatments, and include both bone resorption markers (e.g. type I collagen crosslinked Beta C-telopeptide (βCTX)) and bone formation markers (e.g. type I procollagen-N-propeptide (PINP)). Hypogonadal men with type 2 diabetes have smaller bone size and lower bone turn over^8^. We conducted this analysis to study the effect of soy isoflavone supplementation on BTMs in men with T2DM and low testosterone levels (<12 nmol/L).

## Research Design and Methods

This post-hoc analysis included data from men who had participated in a randomized double blind parallel study investigating the effect of soy isoflavones on testosterone concentrations in men with T2DM^9^. 412 Caucasian male patients with T2DM were screened. 200 men between 45 to 75 years of age with an early morning total testosterone concentrations of less than 12 nmol/L (normal range – 12–25 nmol/L) on at least 2 different occasions with a normal FSH and LH, and a HbA1c of less than 9% (<75 mmol/mol) were recruited. Subjects were randomised either to 15 g soy protein alone (SP) (all isoflavones removed by serial alcohol washing^9^) per day or 15 g soy protein with 66 mg of isoflavones (SPI) per day for 3 months.

Subjects were on stable medications for hypertension, hyperlipidemia and T2DM for at least 3 months prior to the study. Those with significant renal or hepatic impairment, receiving testosterone replacement, who were allergic to soy products, or who had received antibiotics 3 months prior to the study were excluded. Participants were instructed to maintain their level of physical activity throughout the study. Participants were required to avoid food products containing alcohol, soy, alcohol, mineral or vitamin supplementation, and any over-the-counter medications. Plasma isoflavone concentrations were measured at each visit to ensure adherence. All methods were performed in accordance with the relevant guidelines and regulations. All subjects gave their written informed consent. Ethical approval was given by East Yorkshire & North Lincolnshire Research Ethics Committee, ref: 09/H1304/45.

### Study product

Bar containing 7.5 g of the isoflavone-free soy protein (SP) or 7.5 g isolated soy protein powder (Solcon F, Solbar Industries, Ashdod, Israel) with 33 mg of isoflavones (Solgen 40, Solbar Industries Israel) (SPI) was consumed twice daily for 3 months. Essential Nutrition Ltd, Brough, UK performed randomization based on a computer generated randomization list.

### Bone turn over markers

Plasma P1NP and βCTX were measured using an electrochemiluminescent immunoassay (Roche Diagnostics, Lewes, UK). Inter/Intra assay coefficient of variation of plasma βCTX were both <4% between 0.2 and 1.5 µg/L. The assay sensitivity was 0.01 µg/L. Inter/Intra assay CV of plasma P1NP were both <3% between 20–600 µg/L. The assay sensitivity was 8 µg/L.

LC-MS/MS was used to extract and analyse serum phytoestrogens^10^. The inter assay CVs were less than 7.4% for equol, less than 6.1% for genistein and less than 6.8% for daidzein between 0.5 ng/mL to 200 ng/mL. The intra assay CVs were less than 8.0% for equol, less than 3.6% for genistein and less than 7.2% for daidzein between 0.5 ng/mL to 200 ng/mL.

### Statistical analysis

The sample size was initially calculated based on the aims of the primary study^9^. Based on a study using the same soy preparation on bone turnover markers involving post-menopausal women^11^, a post hoc power analysis for changes in βCTX and P1NP using N-query software, for 90% power, 2-tailed with an alpha of 0.05 required 12 subjects per arm for βCTX and 60 subjects per group for P1NP, including a 20% dropout rate.

Baseline continuously distributed data is presented as median (25^th^/75^th^ centiles); categorical data by n (%). Within-group differences (difference between 12 week values and baseline values) are shown for each treatment group separately by a mean and a standard deviation (SD). Between-group comparisons were performed using the independent sample t-test. A two-tailed P < 0.05 was considered to indicate statistical significance for all analysis. Bonferroni corrections were not applied^12^. STATA statistical computer package (StataCorp. 2013. *Stata Statistical Software: Release 13*. College Station, TX: StataCorp LP, USA) was used for statistical analysis.

## Results

412 participants were screened and 200 with T2DM and low testosterone concentrations were recruited. 29 patients dropped out (14 patients SP group and 15 patients SPI group)^9^. The baseline parameters of the two groups are given in Table 1.

**Table 1:** Baseline parameters between the SPI and SP groups.

Parameters	SP1 group (n = 100)	SP group (n = 100)
Age (years)	52.0 (50.0, 55.0)	52.0 (50.0, 55.0)
Weight	100.1(88.5,112.3)	98 (85.7,111.9)
Body Mass Index (kg/m^2^)	31.8 (28.8,34.7)	31.6 (29.2,35.0)
Duration of diabetes (years)	7.3 (4.2,8.8)	7.9 (4.4, 9.1)
HbA1c (mmol/mol)	56 (52,60)	58 (53,64)
TSH (mU/L)	1.6 (1.2, 2.4)	1.6 (1.2, 2.5)
fT4 (pmol/L)	12.0 (12.0, 14.0)	13.0 (12.0, 14.0)
Fasting glucose (mmol/L)	139.5 (118.8, 160.7)	135.9 (115.2, 154.4)
Fasting insulin (µIU/mL)	16.5 (9.9, 25.3)	18.0 (10.4, 28.6)
HOMA-IR	5.6 (3.6, 9.0)	6.2 (3.8, 9.7)
hsCRP (mg/L)	2.1 (0.8, 3.9)	1.9 (0.9, 4.1)
TC (mmol/L)	3.9 (3.4, 4.6)	3.8 (3.4, 4.5)
LDL-C (mmol/L)	2.0 (1.7, 2.9)	2.0 (1.6, 2.7)
HDL-C (mmol/L)	1.1 (0.9, 1.3)	1.0 (0.9, 1.2)
Triglycerides (mmol/L)	1.4 (1.0, 2.1)	1.3 (0.9, 2.0)
Daidzein (ng/mL)	1.4 (0.6, 2.7)	1.9 (0.7, 4.3)
Genistein (ng/mL)	2.6 (0.7, 5.7)	2.9 (1.3, 7.2)
Equol (ng/mL)	0.1 (0.1, 0.1)	0.1 (0.1, 0.1)

SPI (15 g soy protein with 66 mg of isoflavones); SP (15 g soy protein alone without any isoflavones)

Values are provided as medians (25^th^/75^th^ centiles).

To convert values for glucose to milligrams per deciliter, divide by 0.056.

To convert values for insulin to picomoles per liter, multiply by 6.

To convert values for cholesterol to milligrams per deciliter divide by 0.0259.

To convert values for triglycerides to milligrams per deciliter divide by 0.0113.

TC - Total cholesterol; LDL-C - LDL-cholesterol; HDL-C - HDL cholesterol; TG-Triglycerides; HbA1c – glycated hemoglobin; HOMA – Homeostasis model of assessment – insulin resistance; HsCRP – highly sensitive C-respire protein; FSH – follicle stimulating hormone; LH – Luteinizing hormone; SHBG – sex hormone binding globulin; TSH – thyroid stimulating hormone; fT4 – free thyroxine; fT3 – free tri-iodo thyronine.

There was a 15% decrease in βCTX with 3 months of SPI supplementation. There were no changes in P1NP with either SPI or SP supplementation (Table 2). The changes in βCTX with SPI supplementation were significantly greater when compared to three months of SP supplementation.

**Table 2:** Comparison between SPI and SP supplementation at end of study of bone turnover markers.

Parameters	SPI			SP			Difference of the difference p value
	Baseline	3 months	p-value	Baseline	3 months	p value	
	Mean (SD)	Mean (SD)	(3 mo – baseline)	Mean (SD)	Mean (SD)	(3 mo – baseline)	
Bone turn over markers							
βCTX (µg/L)	0.22 (0.09)	0.19 (0.08)	0.04	0.22 (0.10)	0.22 (0.11)	0.89	<0.01
P1NP (µg/L)	31 (12)	32 (11)	0.66	33 (13)	33(12)	0.352	0.322

Paired difference = 3-months-baseline. Difference of the difference is an unpaired t-test of the paired differences.

SPI (15 g soy protein with 66 mg of isoflavones); SP (15 g soy protein alone isoflavone free)

BTM – bone turnover marker.

βCTX - collagen type 1 cross-linked Beta C-telopeptide.

P1NP - propeptide of type I collagen.

There was a significant linear correlation between reduction in βCTX in the SPI group with reduction in HOMA-IR (r^2^ = 0.54; p = 0.02) and HbA1c (r^2^ = 0.42; p = 0.04). There were no correlations between a reduction in βCTX with changes in triglycerides (r^2^ = 0.10; p = 0.48), hsCRP (r^2^ = 0.26; p = 0.33), fT4 (r^2^ = 0.10; p = 0.69) or TSH (r^2^ = 0.12; p = 0.72) concentrations in the SPI group. There was also no correlation between changes in βCTX with SPI supplementation with changes in daidzein (r^2^ = 0.16; p = 0.22), genistein (r^2^ = 0.13; p = 0.54) and equol (r^2^ = 0.20; p = 0.77) concentrations.

## Discussion

In this study, there was a significant reduction in the bone resorption marker βCTX after three months of combined soy protein and isoflavone supplementation in men with T2DM. The reduction in βCTX was correlated with an improvement in glycemic control and insulin resistance suggesting the effect of soy in bone may be mainly through modifying insulin resistance.

Patients with T2DM have increased risk of fractures especially of hip^1,2^. In the Rotterdam Study, an increased fracture risk for nonvertebral fractures (hip and wrist) was described [HR 1.33 (1.00–1.77)] despite higher BMD^13^. In subset analysis, the increased fracture risk seemed to be restricted to treated T2DM [HR 1.69 (1.16–2.46)], with subjects with impaired glucose tolerance showing a reduced fracture risk. This decreased bone strength may develop as a consequence of insulin resistance associated with T2DM and of particular relevance with the finding here of the decrease in βCTX associated with a decrease in insulin resistance. An inverse association of fasting insulin with periosteal circumference has been reported in healthy adolescents^14,15^, and insulin resistance correlated inversely with periosteal and endosteal circumference^16,17^. Other putative indirect mechanisms whereby insulin resistance may affect bone involve a suggested role of hyperinsulinemia in bone ageing^18,19^, reduced blood flow to the bone tissue adversely affecting bone remodeling^19^ and it has been shown that osteoblast-specific disruption of the insulin receptor leads to impaired osteoblast differentiation and reduced trabecular bone formation^20,21^. Experimental studies indicated that similar to skeletal muscle, hepatic, and adipose tissue, insulin resistance can develop in bone tissue, and that this compromised insulin signaling is associated with decreased bone remodeling^22^. Hence improving insulin resistance through soy preparations with a combination of soy protein and isoflavone could potentially improve bone health as shown in this study.

The isoflavone concentrations used in the study preparation reflects the daily intake of Asian population consuming high soy diet or people consuming soy supplements of around 50–90 mg per day^23,24^. Epidemiological studies generally suggest a positive association between soy consumption and BMD^25^. Isoflavones are heterocyclic phenols found in various plant products including soy, the main constituents of which are genistein, daidzein and glycitein. They have a similar structure to 17 beta estradiol and have been shown to have biological activity exerting estrogen-like effects both *in vitro* and *in vivo*. Estrogen can increase BMD and has a protective effect on bones in females. In the present study, there was a mean 15% reduction in βCTX suggesting beneficial effects on bones. These findings are similar to our previous observation in post-menopausal women using similar soy preparations^11^ in which there was a significant reduction in βCTX after 6 months of SPI preparation compared to SP preparation^11^. Potent anti-resorptive agents such as bisphosphonates and denosumab, act by reducing bone turn over although to a greater magnitude^26^. Treatment with bisphosphonate reduce CTX by around 40–50%^27,28^. The reduction of CTX by isoflavones compares well with dietary treatments^11^ and could possibly be used in combination with other anti-osteoporotic drug treatments.

In patients with T2DM circulating biochemical markers of bone formation are decreased^29–31^. In post-menopausal women using a similar soy preparation, changes in P1NP were more pronounced, with a significant decrease in P1NP after 6 months of SPI preparation^11^. In the current study, there were no significant changes in P1NP after 3 months of SPI supplementation that may be due to shorter duration of supplementation.

There were no correlation of changes in βCTX with changes in plasma isoflavones including daidzein, genistein and equol suggesting that the effect of SPI on this bone turnover marker is not a direct effect. This lack of association could also be due to the fact that since the same dose was given to participants, their plasma isoflavones were raised to the same degree but the difference in response to the isoflavones could be due to unmeasured factors such as difference in genetic background and metabolism. There was no correlation of changes in βCTX with changes in lipids or hsCRP, which is an inflammatory marker.

The strengths of this study include that a comparison with a confirmed isoflavone free preparation was undertaken and isoflavone measurement confirmed compliance. Although the study was not powered primarily to look at changes in bone turn over markers, based on another report of bone turnover markers^11^ this study had significant power to detect changes in those markers, accounting for drop outs.

In conclusion, combined soy protein and isoflavone supplementation for three months in men with T2DM significantly reduced the bone-resorption marker βCTX that correlated with an improvement in glycemic control and insulin resistance, suggesting that the effect of soy protein with isoflavones in bone may be mediated through modifying insulin resistance.
